# Predictors of satisfaction after lumbar disc herniation surgery in elderly

**DOI:** 10.1186/s12891-019-2975-4

**Published:** 2019-12-09

**Authors:** Niyaz Hareni, Fredrik Strömqvist, Björn Strömqvist, Björn E. Rosengren, Magnus K. Karlsson

**Affiliations:** 1 Departments of Orthopaedics, Varberg Hospital, Träslövsvägen 68, 432 37 Varberg, Sweden; 2 Departments of Clinical Sciences and Orthopedics, Lund University, Skåne University Hospital, Malmö, Sweden

**Keywords:** Disc herniation, Lumbar, Elderly, Outcome, Surgery

## Abstract

**Background:**

The aim of this study was to evaluate satisfaction and factors associated with satisfaction in elderly undergoing lumbar disc herniation surgery.

**Methods:**

In the national Swedish register for spinal surgery (SweSpine) we identified 2095 patients aged > 65 years (WHO definition of elderly) whom during 2000–2016 had undergone LDH surgery and had pre- and one-year postoperative data (age, gender, preoperative duration and degree of back- and leg pain, quality of life (SF-36) and one-year satisfaction (dissatisfied, uncertain, satisfied). We utilized a logistic regression model to examine preoperative factors that were independently associated with low and high satisfaction and after LDH surgery.

**Results:**

One year after surgery, 71% of the patients were satisfied, 18% uncertain and 11% dissatisfied. Patients who were satisfied were in comparison to others, younger, had shorter preoperative duration of leg pain, higher SF-36 mental component summary and more leg than back pain (all *p* < 0.01). Patients who were dissatisfied were compared to others older, had longer preoperative duration of leg pain and lower SF-36 scores (all *p* < 0.01). 81% of patients with leg pain up to 3 months were satisfied in comparison with 57% of patients with leg pain > 2 years (*p* < 0.001).

**Conclusion:**

Only one out of ten elderly, is dissatisfied with the outcome of LDH surgery. Age, preoperative duration of leg pain, preoperative SF 36 score, and for satisfaction also dominance of back over leg pain, are in elderly factors associated to good and poor subjective outcome after LDH surgery.

## Background

Lumbar disc herniation (LDH) is one cause of sciatica (1), and the disability creates not only individual suffering, but also, with a prevalence of 1–3% (2, 3), also significant costs for society (4). Most LDH patients are treated non-operatively (5), but surgery may be an option, with absolute indication cauda equine syndrome and relative progressive neurological deficits, intractable pain and/or sciatica that do not respond to non-operative management (6). The outcome of surgery is well described in young and middle age adults (7), less in elderly (8, 9). Degenerative spine disease is however common among elderly, and old patients have a lower potential both for recovery after a surgery (8) and for recovery of neurological injuries (10) compared to younger individuals. The factors that associate with outcomes might differ in different age categories. Previous studies have for example shown that preoperative duration of leg pain is associated with outcome in adults (11) but not adolescents (12). Also the implications of gender and smoking should be evaluated in the old patient (11, 13–16) as to facilitate accurate preoperative information and expectations in this patient group. This study therefore aims to describe satisfaction after LDH surgery in elderly and explore preoperative factors independently associated with favorable and poor outcome. We hypothesized that patients aged 65 years or older with longer preoperative duration of leg pain would reach an inferior outcome. We further hypothesized that elderly with more back than leg pain would also have an inferior outcome, due to possible higher incidence of concomitant degenerative spinal changes.

## Methods

The National Swedish Spine Register (SweSpine), a patient centered surgical register that includes patients with degenerative lumbar spine surgery, covers 90% of all departments conducting lower back surgery in Sweden (17) with a completeness of 75% (www.swespine.se). The background and the structure of the register has been reported in detail (18, 19) and validated with adequate results (18–20). The patients report preoperative data on age, gender, smoking and duration of leg pain, categorized in no leg pain, leg pain up to 3 months, 3 to 12 months, 12 to 24 months and more than 24 months. The patients also complete the Numeric Rating Scale (NRS) that estimate back and leg pain (from 0 to 10) and quality of life according to the Short-Form Health Survey 36 (SF-36) (from 0 to 100) in the mental summary component (MCS) (scaled from 0 to 100) and physical summary component (PCS) (from 0 to 100). From the patient reported back and leg pain NRS data, we created a dichotomous preoperative variable, (i) more back than leg pain and (ii) equal or more leg than back pain. Surgeons report surgical data as diagnosis, level of surgery, side, procedure and perioperative complications. The patient reports the one-year follow-up data by letter, equivalent to the preoperative evaluation, but also information on satisfaction with the surgery by use of a Likert Scale (categorized in satisfied, uncertain and dissatisfied).

**Fig. 1:**
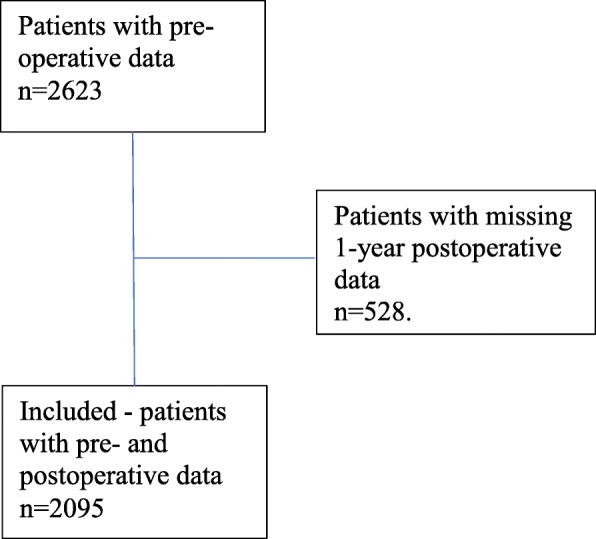
Patient flow chart

We identified in SweSpine between 2000 to 2016, 2623 patients aged 65 years and above with lumbar disc herniation as the index diagnosis and had been addressed by operation with open discectomy with or without microscope (76.9%), decompression (that could include a partial or total laminectomy and/or a root canal decompression) (15.6%), with various other types of surgeries (7.3%) or with type of surgery not reported (0.8%). 528 patients had not participated or answered grade of satisfaction at the one-year follow-up exam (Fig. 1), and were not included in this report. A drop-out table that compared the preoperative data in the 528 with no or missing postoperative data with the 2095 with complete data are presented in Table 1.

**Table 1:** Dropout table, comparing pre-operative data in patients with both pre- and one-year postoperative data (*n* = 2095) with those with no or non-complete one-year postoperative data (*n* = 528). Data are presented as numbers (n), proportions (%), mean ± standard deviation (SD) or mean with 95% confidence intervals (95% CI)

	Patients with pre- and postoperative data *n* = 2095	Patients with no or missing postoperative data n = 528
Age	71.5 ± 5.4	72.1 ± 6.0
Men (%)	52	47
Smokers (%)	10	12
SF-36 PCS	39 (38, 39)	38 (36, 39)
SF-36 MCS	27 (27, 28)	27 (26, 28)
NRS leg pain	7.2 (7.0, 7.6)	6.9 (6.4, 7.7)
Dominant back pain (%)	17	22
Leg pain ≥ back pain (%)	83	78
Duration of leg pain (%)		
0–3 months	15	15
3–12 months	51	48
12–24 months	18	21
> 24 months	16	15

Descriptive data are presented as numbers, means ± standard deviations (SD) or proportions (%). In the first model we compared preoperative data in satisfied patients with uncertain and dissatisfied as to estimate if there are any factors that specially associates with a favorable outcome. In the second model we compared preoperative data in unsatisfied patients with satisfied and uncertain as to estimate if there are any factors that specially associates with a unfavorable outcome. For group comparisons we utilized independent Student’s *t* test between means for continuous data and chi square test for categorical data. Any preoperative factors with a *p*-value below 0.10 was included in a binary logistic regression model to determine independent associations (each variable adjusted for the other variables in the model) as explanatory factor. All variance inflation factors (VIFs) were below 1.1, indicating no multi-collinearity. The Hosmer-Lemeshow test was performed to ensure goodness of fit. We regarded a *p*-value below 0.05 to indicate a statistically significant difference. The study was approved by the Lund regional ethical review board (Dnr 2017/158) and was conducted in accordance with the declaration of Helsinki.

## Results

The 2095 study participants (52% men) had a mean age of 71.5 ± 5.4 years (range 65 to 91). 1495 patients (71%) were satisfied one year after surgery, 380 (18%) uncertain and 220 (11%) dissatisfied. Patients who were satisfied were younger, had a shorter preoperative duration of leg pain, higher SF-36 mental component score and to a greater extent more leg than back pain compared to patients who were uncertain/unsatisfied (all *p* < 0.01) (Table 2). Patients who were unsatisfied, were older, had a longer duration of preoperative leg pain and lower preoperative SF-36 scores than patients who were satisfied/uncertain (all *p* < 0.05) (Table 3).

**Table 2:** Pre-operative data in patients with both preoperative and complete postoperative data (n = 2095), separated in those who become satisfied respectively uncertain or dissatisfied with the surgical outcome Data are presented as numbers (n), proportions (%) or means with 95% confidence intervals (95% CI)

	Satisfied *n* = 1495	Uncertain or dissatisfied *n* = 600	*P*-value
Age	70.6 (70.3, 70.9)	72.2 (71.6, 72.8)	**< 0.001**
Men (%)	53	48	0.06
Smokers (%)	9	11	0.36
SF-36 MCS	28 (27, 28)	26 (26, 27)	**< 0.01**
SF-36 PCS	39 (38, 40)	37 (36, 39)	0.06
NRS leg pain	7.3 (7.0, 7.6)	7.5 (7.0, 8.2)	0.45
Dominant back pain (%)	15	23	**< 0.001**
Leg pain ≥ back pain (%)	85	77	
Duration of leg pain (%)			
0–3 months	17	11	**< 0.001**
3–12 months	54	43	
12–24 months	16	22	
> 24 months	13	25

**Table 3:** Pre-operative data in patients with both preoperative and complete postoperative data (n = 2095), separated in those who become dissatisfied respectively uncertain or satisfied with the surgical outcome Data are presented as numbers (n), proportions (%) or mean with 95% confidence intervals (95% CI)

*n* = 2095	Dissatisfied *n* = 220	Uncertain or satisfied *n* = 1775	*P*-value
Age	72.4 (71.6, 73.3)	70.9 (70.6, 71.2)	**< 0.01**
Men (%)	47	52	0.17
Smokers (%)	10	10	0.84
SF-36 MCS	26 (24, 27)	27 (27, 38)	**< 0.01**
SF-36 PCS	36 (34, 38)	39 (38, 40)	**< 0.05**
NRS leg pain	7.3 (6.9, 7.7)	7.3 (7.0, 7.7)	0.99
Dominant back pain (%)	22	16	0.09
Leg pain ≥ back pain (%)	78	84	
Duration of leg pain (%)			
0–3 months	11	16	**< 0.001**
3–12 months	41	52	
12–24 months	19	18	
> 24 months	29	15	

Among individuals with registered complications, 56% were satisfied, 23% uncertain and 21% dissatisfied compared to 73, 18 and 10% among those with no complication registered (p = < 0.001). Among individuals with open discectomy with or without microscope, 73% were satisfied, 18% uncertain and 10% dissatisfied, compared to 67, 19 and 15% among those with laminar decompression with or without microscope and 69, 21 and 10% in those with various other types of surgeries (*p* = 0.06).

**Fig. 2:**
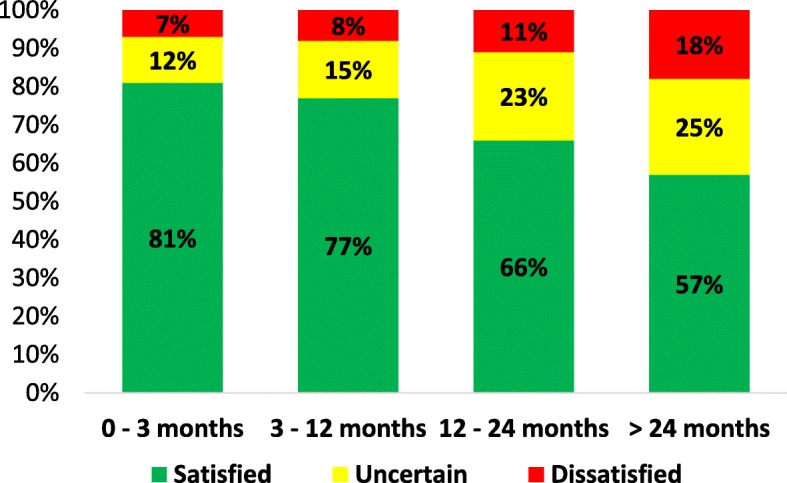
Proportion (%) of satisfied patient one year after surgery in relation to duration of perioperative leg pain. *P*< 0.001 when comparing the four sub-groups with Chi2 with a 4 by 3 table

There was a gradient in the duration of preoperative leg pain, in that with longer duration of leg pain, there was a lower proportion of satisfied patients (*p* < 0.001) (Fig. 2). The post hoc tests revealed similar proportions of satisfied patients in the groups with duration of preoperative leg pain up to 3 and 3 to 12 months (*p* = 0.16). However, all other group comparisons between the different duration categories were statistically significant (Additional file 1: Table S1).

The binary logistic model revealed that age, preoperative duration of leg pain, leg pain dominance over back pain and preoperative SF-36 component were independently associated with a satisfactory outcome (Table 4) and that age, preoperative duration of leg pain and preoperative SF-36 with an unsatisfactory outcome (Table 5).

**Table 4:** Binary logistic regression model with satisfied or uncertain/unsatisfied as dependent variables. Exp (B) for reaching satisfaction is shown per integer for continuous variables

*n* = 1453	Exp (B) with 95 CI	P-value
Age	0.95 (0.93, 0.97)	**< 0.001**
Male	1.1 (0.87, 1.40)	0.44
Female	Reference	
SF-36 MCS	1.03 (1.01, 1.04)	**< 0.01**
SF-36 PCS	1.01 (1.00, 1.02)	**< 0.05**
Dominant back pain	0.63 (0.46, 0.85)	**< 0.01**
Leg pain ≥ back pain	Reference	
Duration of leg pain		
0–3 months	3.22 (2.08, 4.99)	**< 0.001**
3–12 months	2.61 (1.91, 3.57)	**< 0.001**
12–24 months	1.57 (1.08, 2.27)	**< 0.05**
> 24 months	Reference

**Table 5:** Binary logistic regression model with dissatisfied or uncertain/satisfied as dependent variables. Exp (B) for reaching dissatisfaction is shown per integer for continuous variables

n = 1453	Exp (B) with 95 CI	P-value
Age	1.05 (1.02, 1.09)	**< 0.01**
Male	0.99 (0.69, 1.40)	0.94
Female	Reference	
SF-36 MCS	0.97 (0.95, 0.99)	**< 0.01**
SF-36 PCS	0.98 (0.97, 0.997)	**< 0.05**
Dominant back pain	1.37 (0.88, 2.11)	0.16
Leg pain ≥ back pain	Reference	
Duration of leg pain		
0–3 months	0.36 (0.20, 0.68)	**< 0.01**
3–12 months	0.41 (0.27, 0.63)	**< 0.001**
12–24 months	0.49 (0.29, 0.84)	**< 0.01**
> 24 months	Reference	

## Discussion

Our study shows that only one out of 10 elderly patients is dissatisfied with the outcome of LDH surgery. The highest proportion of satisfied patient was seen in the groups operated after no more than 12 months duration of leg pain. Thus, the restitution after nerve root compression post-surgery does not seem worse in elderly compared to middle-aged adults (11, 21–24). We also found that satisfaction, similarly to middle-aged adults, was independently associated with age, SF mental scores and dominant pain location, but in contrast to in middle-aged adults, not with gender or smoking habits (11, 21–24).

We found that age and preoperative SF 36 scores were independently associated with satisfaction. Both age and preoperative mental health impairment have also in middle-aged adults been associated with the outcome of LDH surgery (8, 14). We also found that a larger proportion of patients with preoperative dominant leg over back pain were satisfied with the surgical outcome, compared to patients with equal or more back than leg pain. This also resembles results in middle age, (8, 14), which supports our hypothesis that with less dominant nerve root symptoms and more unspecific back pain, the satisfaction after surgical intervention in elderly is inferior in comparison with dominance of leg rather than back pain the later symptoms indicating more distinct disability due to localized nerve root compression.

The proportion of satisfied patients was higher with shorter duration of leg pain (Fig. 1). For those who underwent LDH surgery after having suffered no more than 12 months leg pain less than 10% were dissatisfied compared to about 20% for those with leg pain more than 24 months. In the binary regression model a duration of leg pain up to 12 months was associated with 3 times greater OR for reaching a satisfactory outcome (Table 4) and 3 times lower OR for reaching a dissatisfactory outcome (Table 5) compared to having more than 24 months of preoperative leg pain, differences that must be regarded as clinically relevant. These results thus oppose our hypothesis that a duration of leg pain up to 3 months would result in the most beneficial outcome. This also indicates that the restitution after nerve root compression after LDH in elderly with relative short duration of symptoms is similar to that of younger adults (8). The reason for the discrepancies in satisfaction when comparing the groups with leg pain duration up to and above 24 months cannot be determined in this study, but we speculate that central sensitization as well as inferior biological restitution capacity might contribute. Another possible explanation factor for these findings might be patient selection bias, one could speculate that the surgeons are more likely to offer operations to the best surgical candidates first and maybe try non-surgical approaches on the patients were the clinical picture is less clear, hence the groups with longer preoperative durations might contain higher proportion of these patients. As this is a hypothesis generating study, we cannot put forward any recommendation on timing of surgery in relation to duration of leg pain, only state that the proportion of satisfied patients were similar in patients with up to 12 months of preoperative leg pain.

Study strengths include the large study population that reflects outcome in a nationwide general health care system including different care givers and surgeons with varying experience and proficiency, and not only highly specialized units with dedicated expert surgeons. Even though the drop out analysis, without obvious differences between participants and non-participants at one-year follow-up, indicates a representable sample, a selection bias for registration may still be possible. Weaknesses include other classical register weaknesses, such as other selection bias, non-randomized study groups, and varying indications for surgery. Another weakness is that we did not have the possibility to re-evaluate the preoperative radiographic examinations. It is therefore likely that there among laminectomized patients actually were some who in addition to a lumbar disc herniation also had spinal stenosis. We can further not exclude that the decompression was performed following per-operative inability to extract disc material due to intense bleeding, scarring or complications. Due to the above-mentioned limitations, the study must be regarded as hypothesis generating inferences of associations rather than causality. Another weakness in Swespine is that the questions used to evaluate duration of back and leg pain have overlapping group limits for 3 (0 to 3 or 3 to 12) and 12 months (3 to 12 or 12 up to 24 months) duration.

## Conclusions

Only one out of ten elderly, is dissatisfied with the outcome of LDH surgery. Age, preoperative duration of leg pain, preoperative SF-36 scores, and for satisfaction also leg pain dominance, are also in elderly factors that on group level are associated with subjective outcome. Gender and smoking habits were, in comparison to as being reported in middle ages adults, not associated with outcome. We found more satisfied patients if duration of preoperative leg pain was no more than 12 months rather than longer.

## Supplementary information

**Additional file 1** **Table S1.** Post-hoc analysis, satisfied or uncertain/dissatisfied analyzed between groups with Chi^2.^

## Abbreviations

EQ-5D: EuroQol 5-dimensions
LDH: Lumbar disc herniation
NRS: Numeric rating scale
ODI: Oswestry disability index
SF-36 MCS: Short-form health survey 36 mental component summary
SF-36 PCS: Short-form health survey 36 physical component summary
SweSpine: National swedish spine register
VIF: Variance inflation factor
WHO: World health orgnanization
