# Supplementary material for: Predictors of satisfaction after lumbar disc herniation surgery in elderly
Source: BMC Musculoskelet Disord. 2019 Dec 9;20:594. doi: 10.1186/s12891-019-2975-4 (PMC6902341; doi:10.1186/s12891-019-2975-4)
Supplement: Supplementary file 1 — Additional file 1 Table S1. Post-hoc analysis, satisfied or uncertain/dissatisfied analyzed between groups with Chi2. [file 12891_2019_2975_MOESM1_ESM.docx]

**Supplementary table 6**

Post-hoc analysis, satisfied or uncertain/dissatisfied analyzed between groups with Chi^2^

|  | **Duration 0-3 months**  **Satisfied: 81 %** | **Duration 3-12 months**  **Satisfied: 77 %** | **Duration 12-24 months**  **Satisfied: 66 %** | **Duration >24 months**  **Satisfied: 57%** |
| --- | --- | --- | --- | --- |
| **Duration >24 months**  **Satisfied: 57%** | **p<0.001** | **p<0.001** | **p=0.03** | **-** |
| **Duration 12-24 months**  **Satisfied: 66 %** | **p<0.001** | **p<0.001** | **-** |  |
| **Duration 3-12 months**  **Satisfied: 77 %** | p=0.16 | **-** |  |  |
| **Duration 0-3 months**  **Satisfied: 81 %** | **-** |  |  |  |
